# Experimental Study on Mechanical Characteristics of Stabilized Soil with Rice Husk Carbon and Calcium Lignosulfonate

**DOI:** 10.3390/ma17215201

**Published:** 2024-10-25

**Authors:** Haiying Zhang, Hongxia Li, Hongze Zhang, Deyue Duan, Qian Ding, Lin Ding, Yanjie Liu

**Affiliations:** 1 School of Civil Engineering, Heilongjiang University, Harbin 150080, China; 2 School of Building Science and Engineering, Nanjing Forestry University, Nanjing 210037, China; dingqian@njfu.edu.cn; 3 College of Engineering and Technology, Northeast Forestry University, Harbin 150040, China

**Keywords:** stabilized silty soil, rice husk carbon, calcium lignosulfonate, microscopic analysis, damage model

## Abstract

In cold regions, the extensive distribution of silt exhibits limited applicability in engineering under freeze–thaw cycles. To address this issue, this study employed rice husk carbon and calcium lignosulfonate to stabilize silt from cold areas. The mechanical properties of the stabilized silt under freeze–thaw conditions were evaluated through unconfined compressive strength tests and triaxial shear tests. Additionally, scanning electron microscopy was utilized to analyze the mechanisms behind the stabilization. Ultimately, a damage model for rice husk carbon–calcium lignosulfonate stabilized silt was constructed based on the Weibull distribution function and Lemaitre’s principle of equivalent strain. The findings indicate that as the content of rice husk carbon and calcium lignosulfonate increases, the rate of improvement in the compressive strength of the stabilized silt progressively accelerates. With an increase in the number of freeze–thaw cycles, the deviatoric stress of the stabilized soil gradually diminishes; the decline in peak deviatoric stress becomes more gradual, while the reduction in cohesion intensifies. The decrease in the angle of internal friction is relatively minor. Microscopic examinations reveal that as the number of freeze–thaw cycles increases, the soil pores tend to enlarge and multiply. The established damage model for stabilized silt under freeze–thaw cycles and applied loads demonstrates a similar pattern between the experimental and theoretical curves under four different confining pressures, reflecting an initial rapid increase followed by a steady trend. Thus, it is evident that the damage model for stabilized silt under freeze–thaw conditions outperforms traditional constitutive models, offering a more accurate depiction of the experimental variations observed.

## 1. Introduction

Silty soil is widely distributed in the northeastern region of China, and the stability of silty soil foundations is crucial to the longevity and safety of infrastructure such as highways and railways. Due to the poor particle bonding characteristics of silty soil [1], it not only experiences substantial cumulative deformation and reduced bearing capacity but is also highly susceptible to liquefaction [2]. Additionally, the hot and rainy summers and the severe cold and snowy winters in northeast China exacerbate these issues [3]. Seasonal freeze–thaw cycles in silty soil subgrades can lead to frost heave and soil heaving, causing significant structural damage and compromising the integrity of the road. Improving the mechanical properties and durability of silty soil subgrades would not only enhance roadway safety and reduce maintenance costs but also extend the lifespan of infrastructure. Therefore, the improvement of silty soil subgrades holds significant theoretical and practical value.

The crux of addressing problems related to silty soil subgrade is based in soil stabilization. This encompasses enhancing the compressive strength of the silty soil, lowering its permeability, and minimizing the damage caused by freeze–thaw cycles. Currently, there is a diverse range of stabilizers for silty soil, primarily categorized into organic, inorganic, and biological enzyme stabilizers [4]. Inorganic stabilizers include cement, fly ash, and lime. However, cement and lime are alkaline materials, and under strongly alkaline conditions, they can significantly impact the reproduction of surrounding flora and fauna [5]. Organic stabilizers include calcium lignosulfonate [6,7], xanthan gum [8], nano-silica, and potassium methyl silicate [9]. Biopolymer stabilizers include microbially induced calcium carbonate using various strains [10,11] and cross-linked biopolymers [12]. When using radiation-induced microbes, it is essential to consider potential hazards to humans and the environment. Therefore, with growing concern over environmental issues, finding new soil stabilizers that are both cost-effective and environmentally friendly has become increasingly important [13,14]. Research indicates that using biomass waste or by-products as soil stabilizers is an effective solution to this problem [15,16].

As China is an agricultural powerhouse, there are abundant rice husk resources with a high annual production [17] in the country. However, utilization rates are low, and large quantities of rice husks are burned, causing severe environmental pollution. Therefore, there is an urgent need to address the large quantities of rice husks piles. Rice husk carbon (RHC) is characterized by its abundant porosity, relatively low density, and large specific surface area. It contains amorphous silica, similar to highly active silica fume [18]. Therefore, rice husk carbon is commonly used as an adsorbent and desiccant [19]. It has also been mentioned in soil improvement applications, where it can enhance properties such as soil water stability [20]. Aziz [21] found that RHC can reduce the swelling properties of expansive soils and enhance their strength. Muhammad’s [22] research indicates that mixing rice husk ash with lime or cement can effectively improve the load-bearing capacity of subgrade soil. Nagaraju et al. [23]. observed that as the amount of rice husk carbon increased during the stabilization of expansive soil, the Free Swelling Index and Plasticity Index decreased, while the California Bearing Ratio and internal friction angle increased, and cohesion decreased. Lignin, a byproduct of bioethanol production, also impacts the natural environment. In engineering applications, lignin is commonly used as a water reducer and binder [24].

Calcium lignosulfonate (CaL) can also serve as a soil stabilizer, enhancing both the cohesion and elastic modulus of the soil [25]. Chen [26] investigated the shear strength and strain hardening behavior of lignin-treated sandy silt through triaxial testing and proposed the reasons for the increase in cohesion from a microstructural perspective. Dong [27] conducted studies using lightweight compaction tests, freeze–thaw cycle triaxial shear tests, thermal constant analysis, and X-ray diffraction tests, varying the lignin fiber content, freeze–thaw cycle frequency, and confining pressure. Chen [28] used lignin as a stabilizer to improve silt, and investigated the changes in shear strength and related parameters of the modified soil through direct shear tests. Kim et al. [29] simulated the pavement performance of lignin-treated subgrade soil. The results indicated that after lignin treatment, the pavement exhibited significantly reduced cracking and rutting depth compared to untreated subgrade, primarily because lignin enhances the elastic modulus and strength of the subgrade soil, thereby improving its pavement performance.

Many scholars both domestically and internationally have produced numerous results on damage models for soil mechanical properties. For instance, Ju [30] investigated the degradation of mechanical properties in landslide shear zones along reservoir banks under wet–dry cyclic conditions. Integrating the Druck–Prager criterion, developing a model that accounts for the compaction characteristics of rocks. Cheng [31] conducted triaxial compression tests and scanning electron microscopy (SEM) experiments on siltstone under different wet–dry cyclic paths and low confining pressures. Based on continuous damage theory and the modified Druck–Prager (D-P) strength criterion, Cheng developed a damage constitutive model for siltstone that accounts for the effects of wet–dry cyclic paths. Deng [32] developed a composite damage model for jointed rock–soil bodies by integrating initial macroscopic and microscopic damage, freeze–thaw damage, and load-induced damage.

Although existing research has found that using biomass materials to stabilize silt is an economical and effective method [33], most studies have focused on the mechanical properties [34] and microscopic characteristic [35,36] of the stabilized silt. Additionally, due to the challenges in ensuring the impact of moisture migration during freeze–thaw cycles [37] and the complex physical properties of silt itself, few researchers have combined damage models with experimental studies of stabilized silt under freeze–thaw conditions [38,39]. Therefore, this study employs rice husk carbon and lignin as eco-friendly stabilizers for silt, determines their optimal ratios, and evaluates their performance under freeze–thaw cycles. Through microscopic analysis, it examines the stabilization mechanisms and develops a damage model that accounts for both freeze–thaw cycles and loading effects. The findings provide valuable insights for enhancing the durability and mechanical performance of silt subgrades in infrastructure projects.

## 2. Materials and Methods

### 2.1. Experimental Materials

#### 2.1.1. Silt

**Figure 1:**
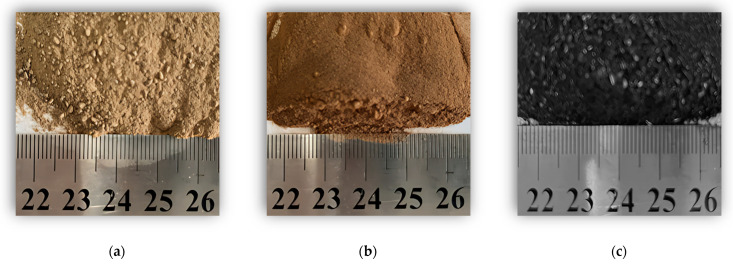
Material: (**a**) Silt, (**b**) Lignin, (**c**) Rice Husk Carbon.

**Figure 2:**
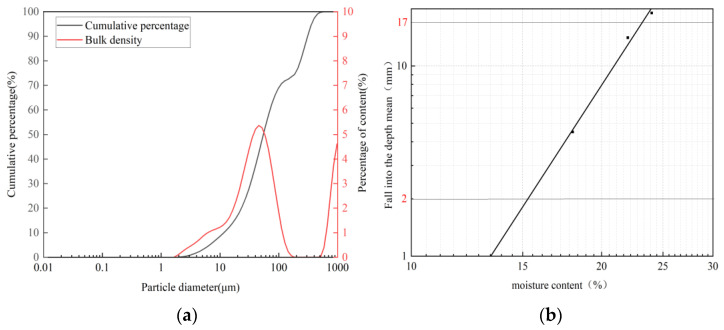
Particle size distribution curve of silt: (**a**) Particle Size Distribution Curve of Silt, (**b**) Liquid Limit and Plasticity Index Curve.

The silt soil was sourced from Harbin, Heilongjiang Province. The soil collected from the field was air-dried naturally, ground into a powder, and sifted through a 2 mm standard sieve, as shown in Figure 1a. According to the “Highway Test Geotechnical Code”, the fundamental physical properties of the soil, including natural moisture content, maximum dry density, and optimal moisture content, were determined (see Table 1). The particle size distribution curve is presented in Figure 2a, and the liquid limit curve is depicted in Figure 2b.

#### 2.1.2. Calcium Lignosulfonate

Calcium lignosulfonate (Figure 1b) is a multi-component polymeric anionic surfactant, commonly known as wood calcium. The lignin content ranges from 40% to 50%. Its chemical formula is C_10_H_24_CaO_10_S_2_. This compound appears as a brownish-yellow powder with a distinct odor and is recognized as a polymeric anionic surfactant [40]; the specific composition of calcium lignosulfonate is detailed in Table 2.

#### 2.1.3. Rice Husk Carbon

Rice husk carbon (Figure 1c) is the product of rice husks burned at 600–700 °C. The carbon content ranges from 45% to 55%. It is black in color and contains various impurities and incompletely burned carbon. After passing through a 2 mm sieve to remove impurities, the specific composition of rice husk carbon is shown in Table 3.

### 2.2. Test Preparation

We prepared the silt to achieve a maximum dry density of 92% and an optimal moisture content of 16.3%. We added rice husk carbon and calcium lignosulfonate in varying percentages. Then, we placed the mixed soil into a mold to create cylindrical samples with a diameter of 39.1 mm and a height of 80 mm. We wrapped the samples in plastic film and stored them in a curing room at a temperature of 24 ± 1 °C and a relative humidity of 98% for 7 days. This curing process allows the SiO_2_ in the rice husk carbon and the calcium lignosulfonate to fully react with the soil minerals, forming a solid soil structure composed of hydrated calcium silicate. The unconfined compressive strength test plan is outlined in Table 4.

We placed the prepared samples in an industrial freezer at −20 °C for 12 h, then thawed them in a water bath at 20 °C for 12 h, completing one freeze–thaw cycle [41]. The number of freeze–thaw cycles was set at 0, 1, 3, 5, and 10. After the designated freeze–thaw cycles were completed, we conducted consolidated undrained triaxial shear tests. The test plan for the freeze–thaw cycles and triaxial shear tests is outlined in Table 5.

### 2.3. Testing Methods

#### 2.3.1. Unconfined Compressive Strength Test (UCS)

The unconfined compressive strength tests were conducted in accordance with the “Standard for Geotechnical Test Methods” (GB/T 50123-2019) using a WDW-100E microcomputer-controlled electronic universal testing machine with a load capacity of 100 kN (Jinan Liangong Testing Technology Co., Ltd., Jinan, China). The testing [42] load rate was set at 1 mm/min. During the loading process, the test was stopped when the compressive strength reached 80% of the peak value, or when the axial strain reached 20%, if no clear peak value was observed.

#### 2.3.2. Triaxial Shear Test

The triaxial shear test was performed on rice husk carbon and lignin-stabilized soil samples that exhibited favorable compressive strength in the unconfined compressive strength test. The shear rate was set at 0.08 mm/min, with confining pressures of 100, 200, 300, and 500 kPa. The test adhered to the consolidated undrained test specifications outlined in the “Standard for Geotechnical Test Methods” (GB/T 50123-2019), using the GDS dynamic and static frozen soil triaxial test system. Sample preparation, maintenance, and freeze–thaw cycles followed established protocols. Before testing, vacuum saturation was performed, followed by back pressure saturation to achieve a saturation level exceeding 95% [43]. The consolidation process was conducted under isotropic conditions.

#### 2.3.3. Scanning Electron Microscope Test

To study the microscopic structural evolution mechanisms of stabilized silt mixed with quantitative rice husk biochar and lignin under the influence of varying freeze–thaw cycles, a ZEISS SUPRA 55 scanning electron microscope (SEM) from Carl Zeiss, Oberkochen, Baden-Württemberg, Germany, was employed to comprehensively analyze the internal mechanisms of the stabilized silt, focusing on the pore changes in the particles. The stabilized silt samples were subjected to 0, 1, 3, 5, and 10 freeze–thaw cycles, followed by cooling and drying to remove moisture. Irregular spheres of 1–2 mm in size were extracted from the samples, and their surfaces were sputter-coated with gold to enhance electrical conductivity before performing SEM analysis. The scanning parameters were as follows: acceleration voltage of 3 kV, working distance of 12.7 mm, and magnification of 5k×.

## 3. Results

### 3.1. Uniaxial Compressive Strength Characteristics

**Figure 3:**
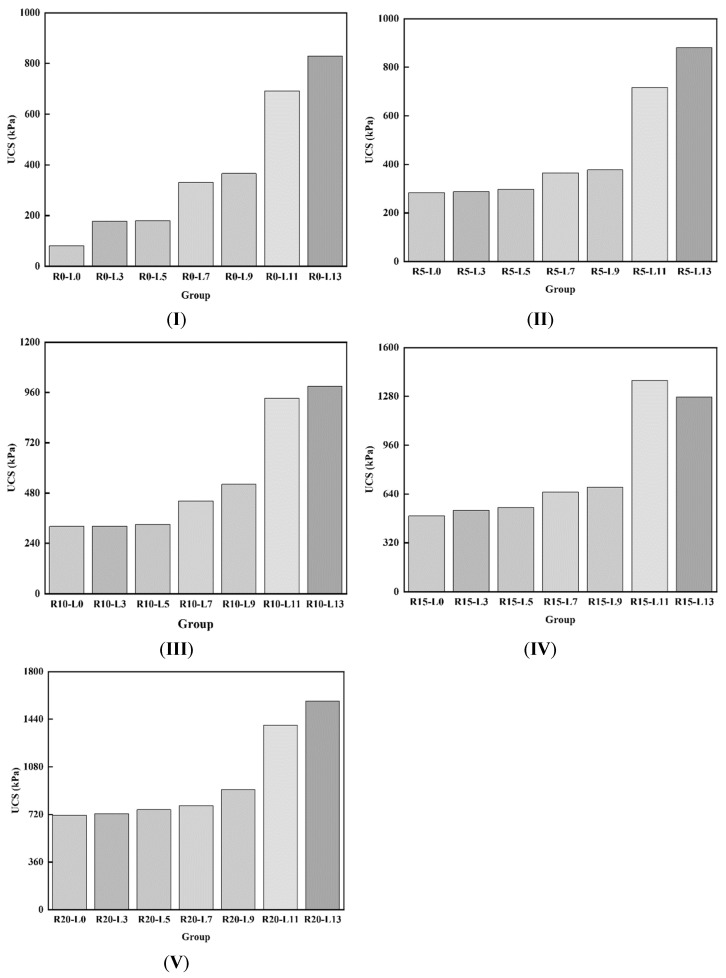
Compressive strength of soil samples with different addition amounts of rice husk carbon and lignin ((**I**)—R0, (**II**)—R5, (**III**)—R10, (**IV**)—R15, (**V**)—R20).

The prepared soil samples were subjected to unconfined compressive strength tests, and the results for the compressive strength of stabilized soil samples with varying amounts of rice husk carbon and calcium lignosulfonate are shown in Figure 3. The data indicate that, with a constant rice husk carbon content, the unconfined compressive strength gradually increases as the lignin content rises. For example, in sample IV, as the lignin content increases, the compressive strength of the samples improves by 7.3%, 11.2%, 31.8%, 38.0%, 179.3%, and 157.5%, respectively. Moreover, when the rice husk carbon content is 15%, the unconfined compressive strength with different lignin ratios is 6.10, 3.01, 3.07, 1.98, 1.87, 2.0, and 1.54 times higher than with 0% rice husk carbon; 1.75, 1.85, 1.85, 1.79, 1.81, 1.94, and 1.45 times higher than with 5% rice husk carbon; and 1.55, 1.66, 1.67, 1.48, 1.31, 1.49, and 1.29 times higher than with 10% rice husk carbon. The rice husk carbon content plays a crucial role in enhancing the unconfined compressive strength of the samples, with the strength increasing as the rice husk carbon content rises. The optimal amount of rice husk carbon is 15%, which yields the most significant strength improvement. Considering economic factors, we selected a cost-effective option while ensuring that the compressive strength meets regulatory requirements. Therefore, soil samples with 15% rice husk carbon and 11% lignin were selected for the freeze–thaw cycle experiment.

### 3.2. Shear Strength Characteristics

#### 3.2.1. Stress–Strain Curve

**Figure 4:**
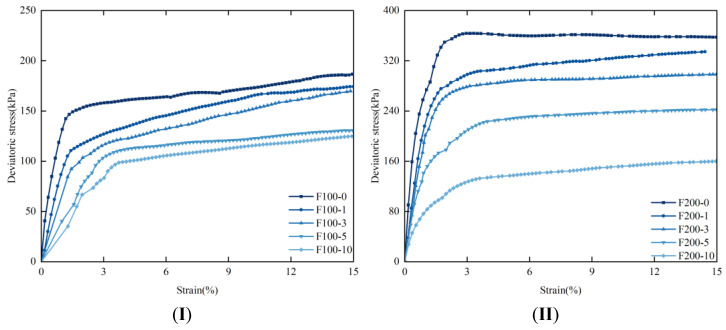

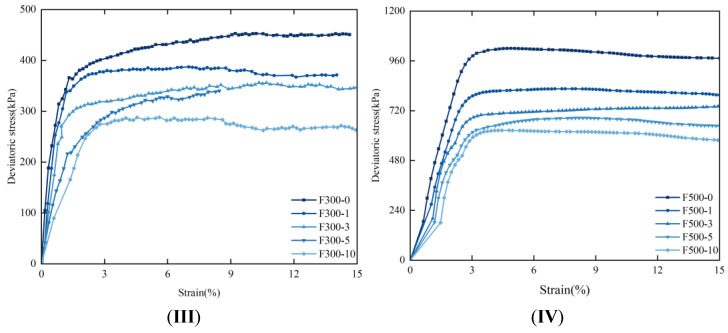
Stress–strain curves of samples under different confining pressures (**I**)—CP = 100 kPa, (**II**)—CP = 200 kPa, (**III**)—CP = 300 kPa, (**IV**)—CP = 500 kPa.

Due to the internal heterogeneity and anisotropy of rice husk carbon–lignin solidified silt soil, the stress–strain curve exhibits significant variability under different confining pressures and various freeze–thaw cycles. The overall transformation can be categorized into the following stages: the elastic stage, the yield stage, and the subsequent strain hardening or strain softening stage. As shown in Figure 4, the stress–strain curves of the specimens under varying confining pressures indicate that, with consistent confining pressure, the peak deviatoric stress gradually increases with the number of freeze–thaw cycles. When the axial strain ranges from 0 to 2.5%, the stress–strain curve rises sharply. At 2.5% axial strain, the peak deviatoric stress stabilizes, as the bonded particles reach their maximum load-bearing capacity. As the number of freeze–thaw cycles and confining pressure increase, the soil undergoes external compression, resulting in a reduction in internal bonding. Consequently, the silt soil’s resistance to external loads progressively diminishes. Interaction and friction between the soil particles contribute to strain hardening, initially strengthening the soil, but ultimately leading to a decline in bearing capacity due to internal damage. These findings underscore the importance of considering freeze–thaw cycles and confining pressure in soil stabilization. While the initial increase in strength is advantageous, the long-term stability of treated soil must be carefully monitored.

#### 3.2.2. Study of the Influence of Freeze–Thaw Cycles on the Elastic Modulus of Stabilized Soil

**Figure 5:**
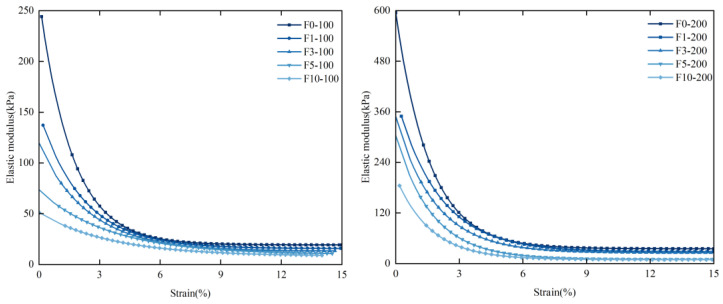

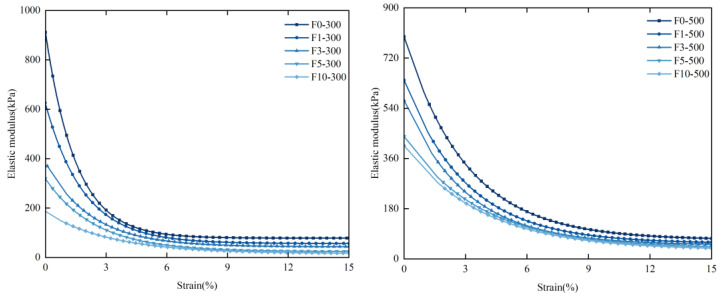
Elastic modulus under different confining pressures and freeze–thaw cycles.

The elastic modulus is a critical parameter that characterizes the mechanical properties of rock and soil. Figure 5 illustrates the elastic modulus of soil samples subjected to consistent confining pressure across various freeze–thaw cycles. The trend in the elastic modulus after multiple freeze–thaw cycles closely mirrors that of unfrozen soil samples. The study reveals an inverse correlation between the elastic modulus and the number of freeze–thaw cycles. As the number of freeze–thaw cycles increases, the elastic modulus consistently decreases, with the rate of decline gradually tapering off. Additionally, as the shear stress rises, the elastic modulus continues to decrease until it stabilizes at a certain threshold. This reduction is attributed to the freeze–thaw cycles disrupting the colloidal particles in the soil and curing agent, resulting in a looser soil structure and diminished strength.

The elastic modulus curves under freeze–thaw cycles at various confining pressures are shown in Figure 5. Confining pressure significantly affects the changes in the elastic modulus of solidified soil. As the number of freeze–thaw cycles increases, the elastic modulus of the solidified soil deteriorates at varying rates. Consequently, Equation (1) can be used to describe the relationship between the elastic modulus E_r_ and the number of freeze–thaw cycles.

(1) $$E_{r}=A+B*e^{-\frac{x}{C}}$$

In this equation, E_r_ represents the elastic modulus, *A*, *B*, and *C* are calculation parameters, and x denotes the number of freeze–thaw cycles.

The experimental data were fitted using the Levenberg–Marquardt optimization algorithm, resulting in the calculation parameters for the variation of elastic modulus with freeze–thaw cycles under different confining pressures, as shown in Table 6.

Analysis of Table 5 shows that the parameters A, B, and C exhibit a linear relationship with confining pressure (CP). Therefore, the relationship can be calculated using Equation (2).

(2) $$D(A,B,C)=f+\frac{\left(e-f\right)}{\left[1+{\left(\frac{x}{i}\right)}^{3}\right]}$$

In this equation, D represents A, B, and C; e and f are parameters. The calculated values for parameters e, f, and i are shown in Table 7.

**Figure 6:**
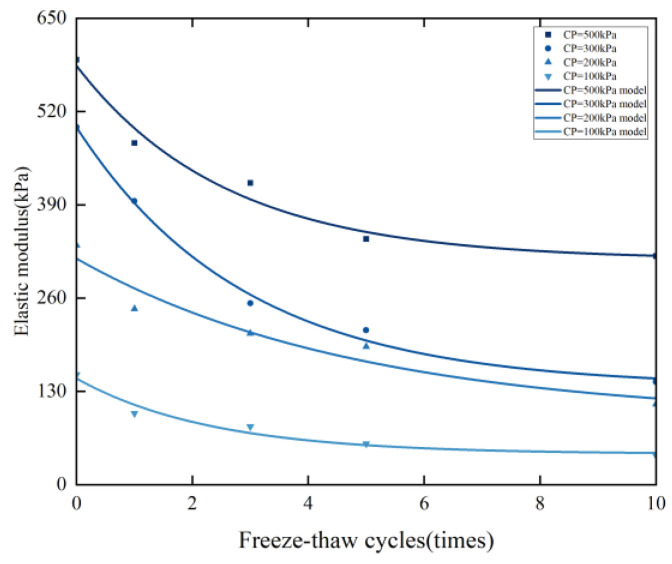
Relationship between cycles and elastic modulus, and the fitting curve of experimental and theoretical calculated values.

Taking confining pressures of 100 kPa, 200 kPa, 300 kPa, and 500 kPa as examples, the experimental data are presented in Figure 6. By substituting the data from Table 5 and Equation (2) into Equation (1), the calculated values are obtained and depicted in the fitting curve in Figure 6. The comparison demonstrates a strong correlation, with experimental values closely matching the calculated ones, achieving a correlation coefficient greater than 0.88. Thus, it is evident that the predictive equations in Equations (1) and (2) effectively describe the relationship between the elastic modulus of solidified silt, the number of freeze–thaw cycles, and the confining pressure.

#### 3.2.3. Changes in Peak Deviatoric Stress, Cohesion, and Internal Friction Angle

**Figure 7:**
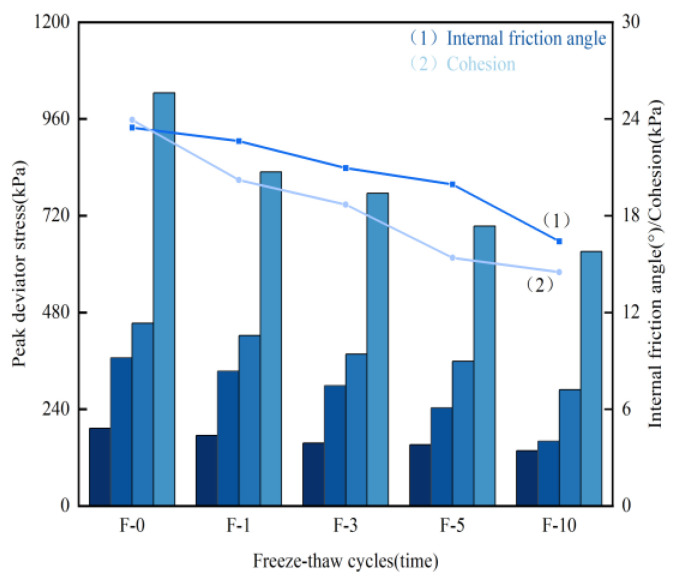
Diagram of peak deviatoric stress, internal friction angle, and cohesion under various confining pressures.

The peak deviatoric stress under various confining pressures is depicted in Figure 7. It is evident that, at the same confining pressure, the peak deviatoric stress of the sample progressively decreases with an increasing number of freeze–thaw cycles. For example, at a confining pressure of 500 kPa, the peak deviatoric stress decreases by 19.18% after one freeze–thaw cycle, 24.43% after three freeze–thaw cycles, 32.28% after five freeze–thaw cycles, and 38.32% after ten freeze–thaw cycles. The rate of decrease in peak deviatoric stress diminishes gradually as the repeated freeze–thaw cycles undermine the internal cementation within the soil, leading to reduced strength and eventual degradation of the soil. These findings underscore the critical importance of considering freeze–thaw cycles in soil stabilization strategies. The significant reduction in peak deviatoric stress emphasizes the need for effective soil treatment methods. Incorporating materials such as rice husk carbon and lignin can mitigate these effects and improve silt soil durability and strength in regions prone to seasonal freeze–thaw cycles.

The peak deviatoric stress measured in this test represents the maximum deviatoric stress on the stress–strain curve after strain hardening has occurred. Peak deviatoric stresses were recorded under confining pressures of 100 kPa, 200 kPa, and 300 kPa. The shear strength parameters of the specimens were calculated using the Mohr–Coulomb failure envelope, as shown in Figure 7, which illustrates the peak deviatoric stress, internal friction angle, and cohesion under each confining pressure. Figure 7(1) displays the variation in internal friction angle with freeze–thaw cycles. As the number of freeze–thaw cycles increases, the internal friction angle of the specimen decreases progressively, with reductions of 3.58%, 10.69%, 15.00%, and 30.08%, respectively. This decrease is attributed to changes in the internal particle bonding mechanism following freeze–thaw cycles, which causes shifts and adjustments in the soil particles stabilized by rice husk carbon–lignin, leading to fluctuations in the internal friction angle. Figure 7(2) depicts the changes in cohesion with freeze–thaw cycles. The figure shows a gradual decline in cohesion as the number of freeze–thaw cycles increases, with reductions of 15.62%, 21.96%, 35.74%, and 39.46%, respectively. Initially, the addition of rice husk carbon–lignin to the silt fills or encases the soil particles. However, as the freeze–thaw cycles progress, the bond between the curing agent and the silt weakens, diminishing the gelling effect and resulting in a gradual reduction in cohesion.

#### 3.2.4. Changes in Damage Strength

**Figure 8:**
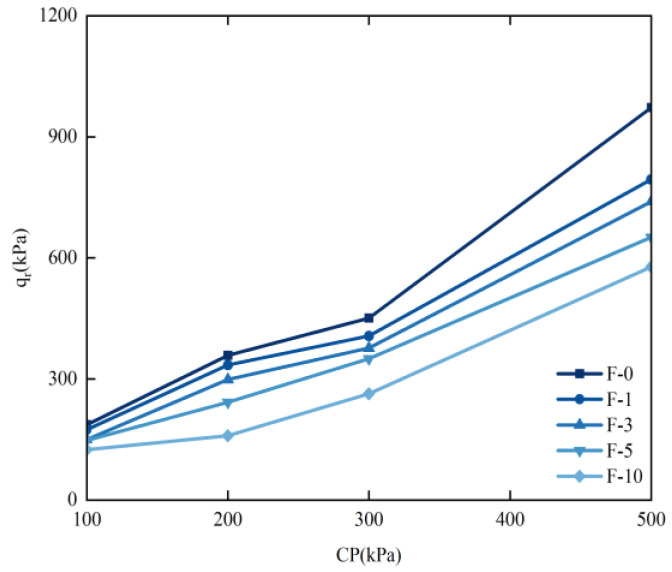
Relationship between residual strength, confining pressure, and number of freeze–thaw cycles.

According to GB/T 50123-2019 “Standard for Geotechnical Test Methods”, the peak point on the σ_1_–σ_3_ curve is identified as the failure point, with the corresponding σ_1_–σ_3_ representing the failure strength q_f_. After specimen failure, the stress gradually stabilizes with increasing strain, and the strength at 15% strain is designated as the residual strength q_r_. The relationship between q_r_, the confining pressure, and the number of freeze–thaw cycles is depicted in Figure 8.

For instance, when the confining pressure is 300 kPa, as the number of freeze–thaw cycles increases, the ratios of the damage strength value to that of the unfrozen soil are 9.8%, 16.47%, 22.34%, and 41.65%, respectively. During the initial freeze–thaw cycles, the limited number of cycles results in minimal changes in damage strength due to the cementation of the soil particles, rice husk carbon, and lignin by water. However, as the freeze–thaw cycles accumulate, the internal cementation within the soil is compromised, reducing its damage resistance and leading to an increased final damage strength compared to the early freeze–thaw stages. These results highlight a significant increase in damage intensity with the number of freeze–thaw cycles, underscoring the necessity of considering freeze–thaw effects in soil stabilization efforts. The minor variations in initial damage strength values suggest that rice husk carbon and lignin enhance soil strength early in the freeze–thaw process. Nonetheless, as the number of cycles grows, the damage intensity eventually rises, indicating the need for more effective soil treatment methods. In regions affected by seasonal freeze–thaw cycles, incorporating materials such as rice husk carbon and lignin can enhance soil durability and strength.

### 3.3. The Mechanism of Calcium Lignosulfonate-Cemented Silty Soil Enhanced with Rice Husk Charcoal

Rice husk carbon creates an alkaline environment that is conducive to the solidification process. In this alkaline milieu, the Ca^2+^ ions from the calcium lignin sulfonate react with SiO_2_ in the soil (as shown in Equations (3) and (4)) to form hydrated calcium silicate colloid (C-S-H), which reinforces the bonds between the soil particles and fortifies the soil structure [44]. Additionally, the calcium lignin sulfonate interacts with the minerals in the silt to form positively charged colloidal ions, as described by reaction equations (such as Equations (5) and (6)), generating hydrated calcium silicate with surface-attached Na^+^ and K^+^ ions. These colloids fill and bind the voids within the soil, effectively restructuring the soil matrix and mitigating deformation during freeze–thaw cycles.

**Figure 9:**
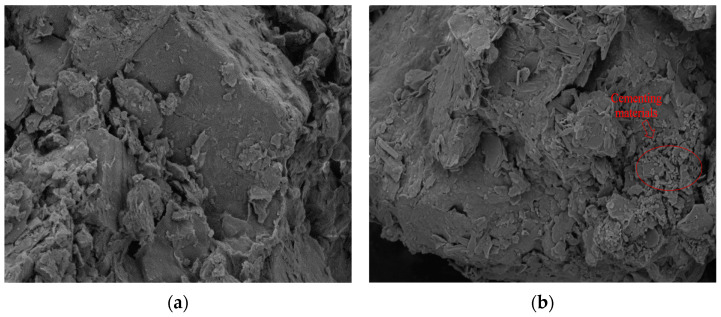
(**a**) Unsolidified soil and (**b**) solidified soil.

As illustrated in Figure 9, the uncured sample (Figure 9a) shows fewer connections between soil particles, with no visible cracks or pores on the surface, resulting in a relatively dense microstructure. In contrast, the sample cured with rice husk carbon and calcium lignin sulfonate (Figure 9b) exhibits flocculent polymers adhering to the soil surface around small pores. This outcome reflects the complete reaction of the curing agent, leading to an uneven distribution of hydrates around the pores, thereby preventing the formation of large pores in the soil sample. This finding is consistent with Liu’s observation [45] that lignin and silt undergo protonation reactions and electrostatic attractions, ultimately forming a dense and stable soil structure. However, in this study, the addition of rice husk carbon further enhances the reaction between the lignin and silt.

(3) $$2OH^{-}+Ca^{2+}=Ca{\left(OH\right)}_{2}$$

(4) $$SiO_{2}+H_{2}O=H_{2}SiO_{3}\;Ca{\left(OH\right)}_{2}+H_{2}SiO_{3}=CaSiO_{3}+2H_{2}O$$

(5) $$Na^{+}-CaSiO_{3}\cdot nH_{2}O+Ca^{2+}\to Ca^{2+}-CaSiO_{3}\cdot nH_{2}O+Na^{+}$$

(6) $$K^{+}-CaSiO_{3}\cdot nH_{2}O+Ca^{2+}\to Ca^{2+}-CaSiO_{3}\cdot nH_{2}O+K^{+}$$

Microstructural analysis of the stabilized soil reveals that rice husk carbon and lignin react with the silt to form hydrated calcium silicate, encapsulating soil particles and filling pores and micro-cracks, thereby enhancing the microstructure of the geopolymer silt matrix. Moreover, the use of rice husk carbon and lignin as materials, rather than traditional alkaline activators, creates a mildly alkaline environment that is more environmentally friendly compared to strong alkaline activators such as NaOH. This approach reduces environmental pollution and mitigates adverse effects on surrounding silt soil and groundwater [46,47]. Therefore, employing agricultural waste materials such as rice husk charcoal and lignin for soil stabilization not only enhances soil properties but also enables rice husk charcoal to adsorb heavy metals like Fe^2+^ and Mn^2+^ from groundwater, thereby mitigating issues of heavy metal contamination in groundwater [48,49].

### 3.4. The Effects of Freeze–Thaw Cycles on Calcium Lignosulfonate-Cemented Silty Soil Enhanced with Rice Husk Charcoal

**Figure 10:**
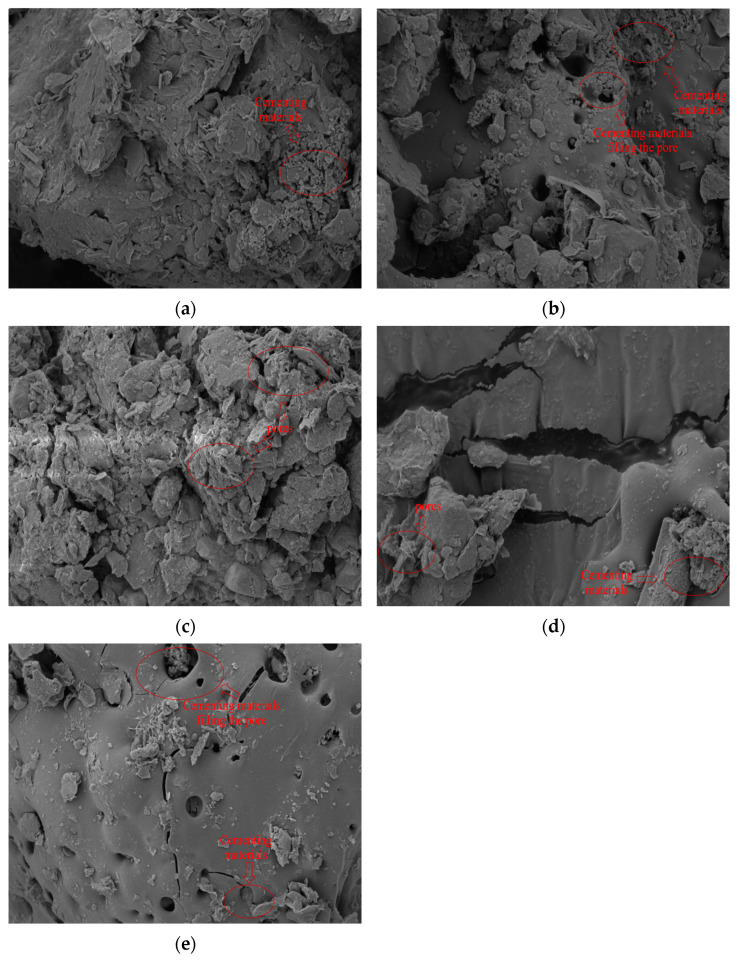
SEM images of rice husk carbon calcium lignin sulfonate stabilized silt after (**a**) 0, (**b**) 1, (**c**) 3, (**d**) 5, and (**e**) 10 freeze–thaw cycles.

After undergoing one freeze–thaw cycle (Figure 10b), the solidified silt soil exhibited some large pores and flocculent polymers on the surface; however, its overall structure remained relatively intact, indicating a degree of freeze–thaw resistance. After 3 and 5 freeze–thaw cycles (Figure 10c,d), the surface of the soil showed a progressive increase in pores and cracks, suggesting that the damage develops gradually rather than suddenly. This gradual deterioration implies that the solidified soil can maintain its structural integrity over a limited range of freeze–thaw cycles. By the time the solidified soil had undergone 10 freeze–thaw cycles (Figure 10e), the repeated cycles caused ice crystals to continuously reshape and grow, disrupting the bonds between the surface polymers and soil particles. The weakening of these bonds and the reduction in the amount of flocculent polymers filling the pores indicate that, even after the ice melts, the polymers cannot fully recover, signifying that the damage inlicted by the freeze–thaw cycles is irreversible [50,51]. Zhu [52] observed that both untreated silt sand and lignin-treated silt sand exhibited dense structures prior to freeze–thaw cycles. However, after undergoing freeze–thaw cycles, the untreated silt sand developed more cracks and pores, resulting in a looser soil structure. In contrast, the lignin-treated silt sand maintained fewer cracks and pores, and its structure remained denser due to the effective encapsulation and filling by the cementitious materials. The rice husk carbon used in this experiment enhances the reaction between the silt and cementitious materials, accelerating the encapsulation and strengthening process, thereby expediting the curing of the solidified soil.

## 4. Damage Model of Solidified Soil Under Freeze–Thaw Cycles

### 4.1. Establishment of Freeze–Thaw Damage Model

After freeze–thaw cycles, the solidified silt soil will exhibit varying degrees of internal damage, including cracks. Assuming that these internal cracks do not change in volume under saturated conditions, the mechanical properties of the solidified silt will deteriorate progressively with an increasing number of freeze–thaw cycles [53]. Therefore, the Weibull distribution function is suitable for describing the internal damage to the rock mass [54]. Let us assume that the probability density function of the micro-soil unit subjected to freeze–thaw cycles is T(F).

(7) $$T\left(F;m,F_{0}\right)={\left(F/F_{0}\right)}^{m-1}\exp\left[-{\left(F/F_{0}\right)}^{m}\right]m/F_{0}$$

In the formula, F represents the strength of the micro-element unit and m denotes the average strength of the micro-element. F_0_ signifies the variation in the elastic modulus of the micro-element soil.

To analyze the damage model of solidified soil subjected to freeze–thaw cycles, let N_f_ be the number of damaged micro-elements after freeze–thaw cycles and N the total number of micro-elements without freeze–thaw cycles, and the microscopic definition of the damage variable D is given by:

(8) $$D=N_{f}/N$$

When a micro-unit is subjected to an intensity range, specifically [F_f_,F_f_ + dF_n_], it will be destroyed, resulting in damage. Consequently, the total number of damaged micro-units is denoted as N_f_.

(9) $$N_{f}=\int_{0}^{F_{f}}NP\left(x\right)dx$$

Combining (6) and (7) gives

(10) $$D=\int_{0}^{F_{f}}NT\left(x\right)dx/N=\int_{0}^{F_{f}}T\left(x\right)dx$$

Combining (5) and (8), we can obtain the damage variable D as

(11) $$D=1-\exp\left[-{\left(F/F_{0}\right)}^{m}\right]$$

The relationship between the damage variable and axial strain of the soil under loading is as follows.

The soil freeze–thaw damage D_F_ can be expressed by the elastic modulus as

(12) $$D_{F}=1{-}_{1}E_{1}/E$$

D_F_ is the damage variable under freeze–thaw cycles, E is the initial modulus, and E_1_ is the modulus after damage.

Given that the state after freeze–thaw damage represents the first damage state, and the damage following loading represents the second damage state, these two damage states are sequentially related [55]. Thus, the total damage variable D_T_ can be expressed as

(13) $$D_{T}=D^{*}+D_{F}-D_{F}D^{*}$$

Combining (10), (11), and (12) gives

(14) $$D_{T}=\left\{ \begin{array}{c} 1-\frac{E_{1}}{E}\exp\left[-{\left(F/F_{0}\right)}^{m}\right],\;\varepsilon_{u}>\varepsilon_{u1}\\ 1-\frac{E_{1}}{E},\;\varepsilon_{u}\leq \varepsilon_{u1} \end{array}\right.$$

In the initial stage of loading, the solidified soil silt is in the elastic stage, and the deformation of the micro-unit of the solidified soil obeys Hooke’s law.

(15) $$\sigma_{1}=E\varepsilon_{u}+\mu \left(\sigma_{2}+\sigma_{3}\right)$$

In the formula, μ represents Poisson’s ratio and E is the initial elastic modulus.

Since this study utilizes conventional triaxial testing conditions where σ_2_ = σ_3_, Equation (14) can be simplified to

(16) $$\sigma_{1}=E\varepsilon_{u}+2\mu \sigma_{3}$$

According to the Lemaitre stress–strain equivalence principle [56,57,58],

(17) $$\varepsilon =\sigma /E_{1}=\sigma^{*}/E$$

(18) $$\sigma^{*}S_{0}=\sigma S_{f}$$

(19) $$D=\left(S_{0}-S_{f}\right)/S_{f}$$

In the equations, σ represents the damaged effective stress, σ* denotes the undamaged equivalent stress, E is the initial modulus, E_1_ is the modulus after damage, S_0_ is the bearing area before damage, and S_f_ is the bearing area after freeze–thaw cycles.

By combining Equations (16), (17) and (19), we obtain

(20) $$\sigma =E\left(1-D\right)\varepsilon_{u}$$

By combining Equations (13) and (19), we obtain

(21) $$\sigma_{1}=\left\{ \begin{array}{c} E_{1}\varepsilon_{u}\exp\left[-{\left(F/F_{0}\right)}^{m}\right]+2\mu \sigma_{3},\;\varepsilon_{u}>\varepsilon_{u1}\\ E_{1}\varepsilon_{u}+2\mu \sigma_{3},\;\varepsilon_{u}\leq \varepsilon_{u1} \end{array}\right.$$

The strength of soil micro-units can accurately describe the evolution of deformation and strength under freeze–thaw cycles. The micro-unit strength F_n_ of the soil can be expressed as follows:

(22) $$F_{n}=F\left(\sigma_{e}\right)$$

In the formula, σ_e_ represents the effective stress of the micro-unit and F(σ_e_) is the stress function.

According to the Mohr–Coulomb criterion,

(23) $$\sigma_{1}-\sigma_{3}\frac{1+\sin\theta }{1-\sin\theta }-\frac{2c\cos\theta }{1-\sin\theta }=0$$

In the formula, c and θ represent the cohesion and internal friction angle. Assuming that the micro-unit strength of the soil follows the Mohr–Coulomb criterion,

(24) $$F_{n}=\sigma_{1}\left(1-\sin\theta \right)-\sigma_{3}\left(1+\sin\theta \right)$$

Combining Equations (15) and (23), we obtain

(25) $$F_{n}=\left(E_{1}\varepsilon_{u}+2\mu \sigma_{3}\right)\left(1-\sin\theta \right)-\sigma_{3}\left(1+\sin\theta \right)$$

Transforming Equation (20) yields

(26) $$\frac{\left(\sigma_{1}-2\mu \sigma_{3}\right)}{E_{1}\varepsilon_{u}}=\exp\left[-{\left(F_{n}/F_{0}\right)}^{m}\right]$$

Further manipulation [59] yields

(27) $$\ln\left\{\ln\left[\left(\sigma_{1}-2\mu \sigma_{3}\right)/\left(E_{1}\varepsilon_{u}\right)\right]\right\}=m\ln\left(-F_{n}\right)-m\ln F_{0}$$

So,

(28) $$Q=\ln\left\{\ln\left[\frac{\left(\sigma_{1}-2\mu \sigma_{3}\right)}{\left(E_{1}\varepsilon_{u}\right)}\right]\right\}$$

(29) $$X=\ln\left(-F_{n}\right)$$

(30) $$A=-m\ln F_{0}$$

From Equations (24) to (29), it follows that

(31) Q=mX+A

The damage model established in this paper adopts Weibull distribution to describe the damage evolution equation of the solidified soil. At this time, the micro-element strength F_n_ is determined by the strength criterion of the rock and soil. By finding the value of F_n_ in the above formula and substituting it into Formula (30), the parameters m and F_0_ are found by fitting curve. The calculated parameters m and F0 are shown in Table 8.

### 4.2. Model Validation

**Figure 11:**
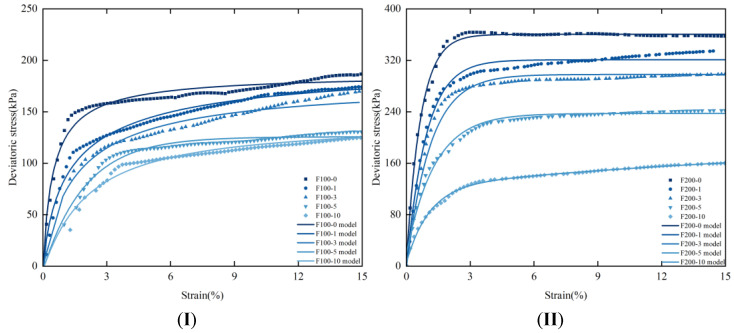

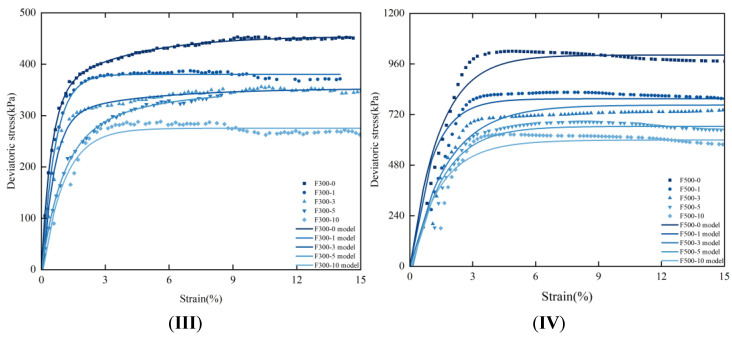
Comparison of model results and test results ((**I**)—CP = 100 kPa, (**II**)—CP = 200 kPa, (**III**)—CP = 300 kPa, (**IV**)—CP = 500 kPa).

The theoretical stress–strain curve derived from the damage model was compared with the experimental curves, as illustrated in Figure 11. The figure reveals that both the experimental and theoretical curves exhibit a similar trend across the four confining pressures, characterized by an initial rapid increase followed by stabilization. The highest degree of fit is observed at a confining pressure of 500 kPa, indicating that the proposed damage model accurately represents the stress–strain behavior of deeply solidified silt. This also demonstrates that higher confining pressures mitigate the damage inflicted by freeze–thaw cycles on soil samples. By integrating continuous damage theory, Weibull distribution theory, and the Mohr–Coulomb strength criterion, a statistical damage model for solidified silt incorporating confining pressure and freeze–thaw cycles has been developed and validated against experimental data.

Drawing on the theories of continuous damage and Weibull distribution, and referencing the Mohr–Coulomb strength criterion, a statistical damage model for cemented silty soil has been developed, incorporating the effects of confining pressure and the number of freeze–thaw cycles. This model is primarily designed to enhance predictions regarding the impact of freeze–thaw cycles on subgrade soil in cold-region engineering. However, it is predominantly suited for strain-hardening curves, and its predictive accuracy for strain-softening curves still requires improvement. Additionally, while the model demonstrates commendable performance under low confining pressure, its predictive capability under high confining pressure remains to be refined.

## 5. Conclusions

In this study, fundamental physical, mechanical, and microscopic tests were conducted to assess the effectiveness of using rice husk carbon and lignin for solidifying silt. The following conclusions were drawn:

1. As the adding of rice husk carbon and lignin content increased, the peak compressive strength of the solidified silt soil improved progressively. For economic considerations, silt samples with 15% rice husk carbon and 13% lignin were selected.
2. Under constant confining pressure, the peak deviatoric stress increased with the number of freeze–thaw cycles. The stress–strain curve exhibited rapid growth when the axial strain ranged from 0% to 2.5%.
3. With more freeze–thaw cycles, the peak deviatoric stress of the soil sample diminished and the range of cohesion significantly decreased, while the internal friction angle remained relatively stable.
4. A statistical damage model for solidified silt, incorporating confining pressure and freeze–thaw cycles, was developed based on continuous damage theory, Weibull distribution theory, and the Mohr–Coulomb strength criterion. The model’s validity was confirmed through experimental data.

## Figures and Tables

**Table 1:** Basic physical properties of silt.

Natural Moisture Content (%)	Liquid Limit (%)	Plastic Limit (%)	Plasticity Index	Optimal Moisture Content (%)	Maximum Dry Density (g/cm^3^)	Uniformity Coefficient	Curvature Coefficient
8.4%	23.2%	15.3%	7.9	16.3%	1.60	6.11	1.32

**Table 2:** Major chemical components of lignin (mass fraction %).

Component	SiO_2_	CaO	MgO	SO_3_	Al_2_O_3_	Fe_2_O_3_	FeO	Loss on Ignition
Content (%)	51.00	3.87	0.93	0.60	32.30	7.56	2.30	1.44

**Table 3:** Chemical composition of rice husk carbon (mass fraction %).

Component	SiO_2_	CaO	MgO	Al_2_O_3_	Fe_3_O_4_	Na_2_O
Content (%)	87.82	2.39	1.34	0.83	0.53	0.27

**Table 4:** Factors and variables for the unconfined compressive strength test.

Group	Category	Rice Husk Carbon Content (%)	Lignin Content (%)
I	R0-L0	0	0
	R0-L3	0	3
	R0-L5	0	5
	R0-L7	0	7
	R0-L9	0	9
	R0-L11	0	11
	R0-L13	0	13
II	R5-L0	5	0
	R5-L3	5	3
	R5-L5	5	5
	R5-L7	5	7
	R5-L9	5	9
	R5-L11	5	11
	R5-L13	5	13
III	R10-L0	10	0
	R10-L3	10	3
	R10-L5	10	5
	R10-L7	10	7
	R10-L9	10	9
	R10-L11	10	11
	R10-L13	10	13
IV	R15-L0	15	0
	R15-L3	15	3
	R15-L5	15	5
	R15-L7	15	7
	R15-L9	15	9
	R15-L11	15	11
	R15-L13	15	13
V	R20-L0	20	0
	R20-L3	20	3
	R20-L5	20	5
	R20-L7	20	7
	R20-L9	20	9
	R20-L11	20	11
	R20-L13	20	13

**Table 5:** Factors and variables for triaxial shear test under freeze–thaw cycles.

Group	Sample	Confining Pressure (kPa)	Freeze–Thaw Cycles
I	F100-0	100	0
	F100-1	100	1
	F100-3	100	3
	F100-5	100	5
	F100-10	100	10
II	F200-0	200	0
	F200-1	200	1
	F200-3	200	3
	F200-5	200	5
	F200-10	200	10
III	F300-0	300	0
	F300-1	300	1
	F300-3	300	3
	F300-5	300	5
	F300-10	300	10
IV	F500-0	500	0
	F500-1	500	1
	F500-3	500	3
	F500-5	500	5
	F500-10	500	10

**Table 6:** Calculation parameters for the variation of elastic modulus with freeze–thaw cycles under different confining pressures.

CP (kPa)	A	B	C	R^2^
500	313.46	270.46	3.37	0.98
300	236.63	261.54	3.29	0.99
200	91.53	223.66	1.45	0.94
100	42.76	105.36	1.43	0.96

**Table 7:** Calculated values for parameters e, f, and i.

Parameter	e	f	i	R^2^
A	105.36	270.46	169.78	0.92
B	42.76	313.46	259.67	0.93
C	1.43	3.37	251.63	0.78

**Table 8:** Calculated parameters m and F_0_.

CP (kPa)		Cycles	0	1	3	5	10
	Parameters						
100	m		0.92	0.85	0.82	0.61	0.58
100	F_0_		1339.43	1450.98	1510.2	897.85	1043.15
200	m		0.97	0.91	0.93	0.94	0.92
200	F_0_		1603.5	2059.05	1635.98	1465.5	1236.45
300	m		0.930.74	0.91	0.74	0.83	0.70
300	F_0_		2208.35	2440.60	2164.62	2075.58	2514.93
500	m		0.76	0.81	0.67	0.63	0.54
500	F_0_		2275.60	2751.7	2230.54	3133.79	2514.93

## Data Availability

The data used to support the findings of this study are included within the article.
